# Tripartite entropic uncertainty relation under phase decoherence

**DOI:** 10.1038/s41598-021-90689-3

**Published:** 2021-06-04

**Authors:** R. A. Abdelghany, A.-B. A. Mohamed, M. Tammam, Watson Kuo, H. Eleuch

**Affiliations:** 1 Physics Department, Faculty of Science, Al-Azhar University, Assiut, 71524 Egypt; 2 Department of Mathematics, College of Science and Humanities, Prince Sattam Bin Abdulaziz University, Al-Aflaj, Saudi Arabia; 3 Department of Mathematics, Faculty of Science, Assiut University, Assiut, 71516 Egypt; 4 Department of Physics, National Chung Hsing University, Taichung, 402 Taiwan; 5 Department of Applied Physics and Astronomy, University of Sharjah, Sharjah, 27272 United Arab Emirates; 6 Department of Applied Sciences and Mathematics, College of Arts and Sciences, Abu Dhabi University, Abu Dhabi, 59911 United Arab Emirates; 7 Institute for Quantum Science and Engineering, Texas A&M University, College Station, TX 77843 USA

**Keywords:** Quantum information, Qubits

## Abstract

We formulate the tripartite entropic uncertainty relation and predict its lower bound in a three-qubit Heisenberg XXZ spin chain when measuring an arbitrary pair of incompatible observables on one qubit while the other two are served as quantum memories. Our study reveals that the entanglement between the nearest neighbors plays an important role in reducing the uncertainty in measurement outcomes. In addition we have shown that the Dolatkhah’s lower bound (Phys Rev A 102(5):052227, 2020) is tighter than that of Ming (Phys Rev A 102(01):012206, 2020) and their dynamics under phase decoherence depends on the choice of the observable pair. In the absence of phase decoherence, Ming’s lower bound is time-invariant regardless the chosen observable pair, while Dolatkhah’s lower bound is perfectly identical with the tripartite uncertainty with a specific choice of pair.

## Introduction

In quantum mechanics, the precise instantaneous measurement of two incompatible observables of a quantum system is generally limited by Heisenberg’s uncertainty principle, in which a lower bound in measurement accuracy is given^1^. After Heisenberg’s work, there have been several attempts to formulate the uncertainty principle in a more comprehensive manner. As an example, Kennard and Robertson^2,3^ proposed an uncertainty principle for the standard deviation for an arbitrary quantum state $|\psi \rangle$ as follows

1 $$\begin{aligned} \Delta X\Delta Z \ge {1 \over 2}\left| {\left\langle \psi \right| [X,Z]\left| \psi \right\rangle } \right| , \end{aligned}$$

where $\Delta X$ and $\Delta Z$ are the standard deviations of the incompatible observables *X* and *Z*, respectively. The uncertainty lower bound given by the right-hand side (r.h.s) of Eq. (1) depends on the quantum state under inspection $\left| \psi \right\rangle$, and becomes trivial ($=0$) when $\left| \psi \right\rangle$ is one of the eigenstates of *X* or *Z*. In contemporary quantum information, the standard deviation in the uncertainty principle is usually reformulated by Shannon entropy in the so-called entropic uncertainty relation (EUR)^4,5^. The EUR of arbitrary two incompatible observables, *X* and *Z* takes the form:

2 $$\begin{aligned} H(X) + H(Z) \ge {\log _2}{1 \over c} \equiv {q_{\mathrm{{MU}}}}, \end{aligned}$$

where $H(V) = - \sum \nolimits _v p (v){\log _2}p(v)$ is the Shannon entropy of observable *V* $(V \in (X,Z))$; *p*(*v*) is the probability for the measurement outcome *v*. *c* is the maximal complementarity between *X* and *Z*, defined by $c = {\max _{\{ l,m\} }}{\left| {\left\langle {{x_l}\left| {{z_m}} \right\rangle } \right. } \right| ^2}$, where $| x_l \rangle$ and $| z_m \rangle$ are eigenvectors of *X* and *Z*, respectively. The benefit of expression (2) is evident that the lower bound only relates to measured observables, i.e., a state-independent one rather than that given by Eq. (1).

The desire to obtain more accurate measurements led to further improvements in the uncertainty relation. Significant progress has been made in this regard recently by Berta et al.^6^, by considering a bipartite system, A and B, in which one (A) is under measurement while the other (B) works as a quantum memory. The quantum-memory-assisted entropic uncertainty relation (QMA-EUR) reads

3 $$\begin{aligned} S(X|B ) + S(Z|B) \ge S(A|B) + {q_{\mathrm{{MU}}}}, \end{aligned}$$

where $S(V|B) = S(\rho _{VB})-S(\rho _{B})$ $(V \in (X,Z))$ is the conditional von Neumann entropy of the measurement outcomes (after *V* is measured) with $S(\rho ) = - Tr[\rho \log _2 \rho ]$ for some density matrix, $\rho$. $\rho _{AB}$ and $\rho _{B}$ are the density matrices for the whole system and subsystem B. The density matrices of post-measurement states are

4 $$\begin{aligned} \rho _{XB}= & {} \sum \nolimits _i ( {| {x _i } \rangle \langle {x _i } | \otimes \mathbf{I}})\rho _{AB} ( {| {x _i } \rangle \langle {x _i } | \otimes \mathbf{I}}),\nonumber \\ \rho _{ZB}= & {} \sum \nolimits _i ( {| {z _i } \rangle \langle {z _i } | \otimes \mathbf{I}})\rho _{AB} ( {| {z _i } \rangle \langle {z _i } | \otimes \mathbf{I}}) \end{aligned}$$

Surprisingly, in the scenario that two observers, Alice and Bob may respectively conduct the measurements on subsystems A and B, the QMA-EUR states that the measurements may become less uncertain if A and B are entangled, i.e., $S(A|B) <0$. By observing his own subsystem B, Bob can predict the result of Alice’s measurement on subsystem A, not to mention that the lower bound of uncertainty becomes zero when the subsystems are maximally entangled^7^.

The lower bound of the QMA-EUR was recently made tighter by Adabi et al.^8^:

5 $$\begin{aligned} S(X|B ) + S(Z|B) \ge S(A|B) + {q_{\mathrm{{MU}}}}+\max \{ 0,\delta \}, \end{aligned}$$

where a term $\delta = I(\rho _{AB} ) - [I(\rho _{XB} ) + I(\rho _{ZB} )]$ is added. $I(\rho _{AB} )$ is the mutual information of A and B, while

6 $$\begin{aligned} I(\rho _{VB} ) = S(\rho _B ) - \sum \limits _i {p_i } S(\rho _i^B ),\;\;\;\;V \in \{ X,Z\} \end{aligned}$$

is the Holevo quantity, which determines the maximum amount of the accessible information about the observable *V*. Here, when Alice measures the observable *V* on the part A, she will obtain an *i*-th outcome $v_i$ with a probability $P_{v_i} = Tr_{AB} [\Pi _i^A \rho _{AB} \Pi _i^A ]$, and the corresponding state of Bob will be transformed into $\rho _i^B = {{Tr_B [\Pi _i^A \rho _{AB} \Pi _i^A ]} \over {P_{v_i} }}$.

These new formulations of the uncertainty principle have facilitated the emergence of many potential applications in the field of quantum information, including cryptography^9,10^, quantum metrology^11,12^, quantum randomness^13,14^ and entanglement witness^15–18^. Much interest has been focused on clarifying how the QMA-EUR evolves in various systems including Heisenberg spin models^19–28^, Unruh–DeWitt detector model^29,30^, neutrino oscillations^31^ and some open quantum systems^21,31–35^.

Renes and Boileau^36^ presented the tripartite version of the QMA-EUR, which can be explained by a guessing game (called the monogamy game) played by Alice, Bob, and Charlie, who share a tripartite quantum state ${\rho _{ABC}}$. At first, Alice measures her part A by choosing one of the observables *X* and *Z*, and obtains an outcome $\kappa$ . Then, informed by Alice with which observable she has measured, Bob and Charlie try to reduce their doubt about Alice’s result as they win when correctly guess her measurement outcome $\kappa$. In this scenario, the tripartite QMA-EUR takes the form,

7 $$\begin{aligned} S(X|B ) + S(Z|C) \ge {q_{\mathrm{{MU}}}}, \end{aligned}$$

in which the lower bound of uncertainty remains the same regardless any change in the prepared state ${\rho _{ABC}}$ since ${q_{\mathrm{{MU}}}}$ only depends on the complementarity between *X* and *Z*.

Very recently, Ming et al.^37^ introduced a tighter bound of the tripartite QMA-EUR by adding some terms related to mutual information and Holevo quantity as follows:

8 $$\begin{aligned} S(X|B) + S(Z|C) \ge {q_{\mathrm{{MU}}}} + \max \{ 0,\Delta \} , \end{aligned}$$

in which

9 $$\begin{aligned} \Delta= & {} {q_{\mathrm{{MU}}}} + 2S({\rho ^A}) - [I(A:B) + I(A:C)]\nonumber \\&+ [I(Z:B) + I(X:C)] - [H(X) + H(Z)], \end{aligned}$$

where the mutual information *I*(*X* : *C*) and the Holevo quantity *I*(*Z* : *B*) are the same as those in Eq. (5), and Shannon entropy *H*(*X*) (*H*(*Z*)) of *X* (*Z*) measurement. In the same context, Dolatkhah et al.^38^ have proposed another lower bound of the tripartite uncertainty that is tighter than that suggested by Ming et al. (8) for some states ${\rho _{ABC}}$. Their formula reads

10 $$\begin{aligned} S(X|B) + S(Z|C) \ge {q_{\mathrm{{MU}}}} + {{S(\left. A \right| B) + S(\left. A \right| C)} \over 2} + \max \{ 0,\delta \} , \end{aligned}$$

in which

11 $$\begin{aligned} \delta = {{[I(A:B) + I(A:C)]} \over 2} - [I(X:B) + I(Z:C)]. \end{aligned}$$

In this study, we investigate the dynamical behavior of the tripartite QMA-EUR and its relations to the nearest- and next-nearest-neighbor entanglement in a three-qubit Heisenberg XXZ chain with decoherence. Also, we introduce a comparative study between the lower bound of Ming et al. and that of Dolatkhah et al., as well as how they depend on the the choice of incompatible observables.

This paper is arranged as follows: in “Heisenberg model with phase decoherence and its solution”, the theoretical model with phase decoherence and its solution is introduced. The tripartite QMA-EUR, Ming’s bound, and Dolatkhah’s bound of the proposed model are reported in “The tripartite QMA-EUR” for two different pairs of the incompatible observables. “Conclusions” contains a concise conclusion of our results.

## Heisenberg model with phase decoherence and its solution

The equation that governs the dynamics of a quantum system described by Hamiltonian *H* with phase decoherence can be obtained by applying the superoperator ${\hat{L}}$ to the density matrix $\rho$ as follows

12 $$\begin{aligned} {{d\rho } \over {dt}} = {\hat{L}}\rho (t) = - i[H,\rho (t)] - {\gamma \over 2}[H,[H,\rho (t)]], \end{aligned}$$

where $\gamma$ is the phase decoherence rate. The system under consideration is a three-qubit Heisenberg XXZ chain in a uniform magnetic field *B*:

13 $$\begin{aligned} H = \sum \limits _{n = 1}^2 {J(\sigma _n^x\sigma _{n + 1}^x + \sigma _n^y\sigma _{n + 1}^y) + {J_z}\sigma _n^z\sigma _{n + 1}^z} + B \sum \limits _{n = 0}^2 \sigma _{n + 1}^z, \end{aligned}$$

where $\sigma _n^\alpha (\alpha = x,y,z)$ are Pauli spin matrices for qubit *n*. *J* and $J_z$ are the spin coupling strengths that we may set $J=1$ without loss of generality. Our choice of the Heisenberg XXZ spin chain model is justified by the fact that it has quantum simulation applications^39–41^. Furthermore, the XXZ model in a transverse field was recently constructed using magnetic atoms on a surface^42^, proposing that the quantum correlations in such models can be measured experimentally.

The solution of Eq. (12) is

14 $$\begin{aligned} \rho (t) = {e^{{\hat{L}}t}}\rho (0), \end{aligned}$$

where $\rho (0)$ is the initial density matrix of the system under consideration, and can be expressed as a linear combination of the eigenstates $\{ | {{\psi _m}} \rangle \}$ as $\rho (0) = \sum \nolimits _{mn} {{\alpha _{mn}}} | {{\psi _m}} \rangle \langle {{\psi _n}} |$. Eigenvectors $|{\psi _m}\rangle$ and the associated eigenvalues $E_m$ satisfy the eigenvalue equations for *H*, $H| {{\psi _m}} \rangle = {E_m}| {{\psi _m}} \rangle (m = 1,2,\ldots 8)$. Therefore, the operation of ${{\hat{L}}}$ on $| {{\psi _m}} \rangle \langle {{\psi _n}} |$ is given by,

15 $$\begin{aligned} {\hat{L}}| {{\psi _m}} \rangle \langle {{\psi _n}} |= & {} - i({E_m}| {{\psi _m}} \rangle \langle {{\psi _n}} | - | {{\psi _m}} \rangle \langle {{\psi _n}} |{E_n})\nonumber \\&- {\gamma \over 2}(E_m^2| {{\psi _m}} \rangle \langle {{\psi _n}} | - 2{E_m}| {{\psi _m}} \rangle \langle {{\psi _n}} |{E_n} + | {{\psi _m}} \rangle \langle {{\psi _n}} |E_n^2) \end{aligned}$$

In turn, the temporal evolution of $\rho (0)$ takes the form:

16 $$\begin{aligned} \rho (t) = \sum \limits _{m,n = 1}^8 {{\alpha _{mn}}{\beta _{mn}}| {{\psi _m}} \rangle \langle {{\psi _n}} |} , \end{aligned}$$

in which

17 $$\begin{aligned} {\beta _{mn}} =\exp ( - it({E_m} - {E_n}) - {{\gamma t} \over 2}{({E_m} - {E_n})^2}) \end{aligned}$$

Equation (16) gives a general description of the tripartite state shared by the three qubits for a given time *t*, ${\rho _{ABC}}(t)$, while the reduced density matrix of any two of the qubits can be obtained by tracing out the other one.

We consider the initial state of the system to be $| {\varphi (0)} \rangle = {1 \over {\sqrt{2} }}(| {000} \rangle + | {110} \rangle )$ to determine the tripartite entropic uncertainty relation and its robustness when the measured qubit is maximally entangled with one of the quantum memories. According to this initial state the measured qubit *A* and the quantum memory *B* are initially in the maximally entangled state $| {\varphi (0)} \rangle = {1 \over {\sqrt{2} }}(| {00} \rangle + | {11} \rangle )$ and the other quantum memory *C* is initially in $| {0} \rangle$. Using the notation of basis $\{ | {000} \rangle ,| {001} \rangle ,| {010} \rangle ,| {011} \rangle , | {100} \rangle ,| {101} \rangle ,| {110} \rangle ,| {111} \rangle \}$, ${\rho _{ABC}}(t)$ can be written as

18 $$\begin{aligned} {\rho _{ABC}}(t) =\left( \begin{array}{cccccccc} A_{11}&{}0&{}0&{}A_{14}&{}0&{}A_{16}&{}A_{17}&{}0 \\ 0&{}0&{}0&{}0&{}0&{}0&{}0&{}0 \\ 0&{}0&{}0&{}0&{}0&{}0&{}0&{}0 \\ A_{41}&{}0&{}0&{}A_{44}&{}0&{}A_{46}&{}A_{47}&{}0 \\ 0&{}0&{}0&{}0&{}0&{}0&{}0&{}0 \\ A_{61}&{}0&{}0&{}A_{64}&{}0&{}A_{66}&{}A_{67}&{}0 \\ A_{71}&{}0&{}0&{}A_{74}&{}0&{}A_{76}&{}A_{77}&{}0 \\ 0&{}0&{}0&{}0&{}0&{}0&{}0&{}0 \\ \end{array} \right) , \end{aligned}$$

and the non-vanishing elements are:

19 $$\begin{aligned} {A_{14}}= & {} {{ - {e^{ - 2t(2B + {J_z})(\gamma (2B + {J_z}) + i)}}} \over 4} + \sum \limits _{ \odot = + , - } {{{2{e^{ - {1 \over 2}t(4B + 3{J_z} \odot \eta )(\gamma (4B + 3{J_z} \odot \eta ) + 2i)}}} \over {\Delta _ \odot ^2}}},\nonumber \\ {A_{16}}= & {} \sum \limits _{ \odot = + , - } {{{ - {\delta _ \odot }{e^{ - {1 \over 2}t(4B + 3{J_z} \odot \eta )(\gamma (4B + 3{J_z} \odot \eta ) + 2i)}}} \over {\Delta _ \odot ^2}}},\nonumber \\ {A_{17}}= & {} {{{e^{ - 2t(2B + {J_z})(\gamma (2B + {J_z}) + i)}}} \over 4} + \sum \limits _{ \odot = + , - } {{{2{e^{ - {1 \over 2}t(4B + 3{J_z} \odot \eta )(\gamma (4B + 3{J_z} \odot \eta ) + 2i)}}} \over {\Delta _ \odot ^2}}},\nonumber \\ {A_{44}}= & {} {{2{e^{ - 2{\eta ^2}\gamma t}}\cos (2\eta t) + J_z^2 + 6} \over {4{\eta ^2}}} + \sum \limits _{ \odot = + , - } {{{ - {e^{ - (4 + {J_z}{\delta _ \odot })\gamma t}}\cos ({\delta _ \odot }t)} \over {8 + {J_z}{\delta _ \odot }}}},\nonumber \\ {A_{46}}= & {} {{{J_z}} \over {4{\eta ^2}}} + \sum \limits _{ \odot = + , - } {{{{\delta _ \odot }} \over 2}} \left( {{{{e^{ - {1 \over 2}t{\delta _ \odot }(2i + \gamma {\delta _ \odot })}}} \over {\Delta _ \odot ^2}} - {{{e^{ - 2t\eta ( \odot i + \gamma \eta )}}} \over {4{\eta ^2}}}} \right) ,\nonumber \\ {A_{47}}= & {} {{{e^{ - 2{\eta ^2}\gamma t}}\cos (2\eta t) - 1} \over {2{\eta ^2}}} + \sum \limits _{ \odot = + , - } {{{i{e^{ - (4 + {J_z}{\delta _ \odot })\gamma t}}\sin ({\delta _ \odot }t)} \over {8 + {J_z}{\delta _ \odot }}}},\nonumber \\ {A_{66}}= & {} {{1 - {e^{ - 2{\eta ^2}\gamma t}}\cos (2\eta t)} \over {8 + J_z^2}},\nonumber \\ {A_{67}}= & {} {{{J_z}} \over {4{\eta ^2}}} - \sum \limits _{ \odot = + , - } {{{{\delta _ \odot }} \over 2}} \left( {{{{e^{ - {1 \over 2}t{\delta _ \odot }(2i + \gamma {\delta _ \odot })}}} \over {\Delta _ \odot ^2}} - {{{e^{ - 2t\eta ( \odot i + \gamma \eta )}}} \over {4{\eta ^2}}}} \right) ,\nonumber \\ {A_{77}}= & {} {{2{e^{ - 2{\eta ^2}\gamma t}}\cos (2\eta t) + J_z^2 + 6} \over {4{\eta ^2}}} + \sum \limits _{ \odot = + , - } {{{{e^{ - (4 + {J_z}{\delta _ \odot })\gamma t}}\cos ({\delta _ \odot }t)} \over {8 + {J_z}{\delta _ \odot }}}},\nonumber \\ {A_{11}}= & {} {1 \over 2},\quad \quad \quad {A_{41}} = A_{14}^*,\quad \quad {A_{61}} = A_{16}^*,\quad \quad {A_{64}} = A_{46}^*,\nonumber \\ {A_{71}}= & {} A_{17}^*,\quad \quad {A_{74}} = A_{47}^*,\quad \quad {A_{76}} = A_{67}^* \end{aligned}$$

where $$\eta = \sqrt{8 + J_z^2} $$, $${\delta _ \pm } = ({J_z} \pm \eta )$$, and $${\Delta _ \pm } = \sqrt{8 + \delta _ \pm ^2} $$.

## The tripartite QMA-EUR

Equations (18) and (19) enable us to get the tripartite entropic uncertainty denoted by $\mathcal{U}$, and lower bounds of Ming et al. (8) and Dolatkhah et al. (10), which will be denoted in this study as $U_M$ and $U_D$, respectively. The lower bounds require the calculation of the post-measurement states referred to which pair of incompatible measurements have been performed on qubit A. To show the dependence of the QMA-EUR on the choice of that pair, we will consider two different cases, $( X , Z )=$($\sigma _x$, $\sigma _z$) and ($\sigma _x$, $\sigma _y$).

### QMA-EUR for ($\sigma _x$, $\sigma _z$)-measurement pair

First we consider the case that Alice measures one of the pair ($\sigma _x$, $\sigma _z$) on her quantum system. If $\sigma _x$ is her choice, then Bob has to guess her outcome. If she chooses $\sigma _z$, Charlie will take the turn. Thereby, one could derive the post-measurement states and the corresponding entropies for the two choices and those of the reduced matrices ${\rho _B} = t{r_{AC}}({\rho _{ABC}})$ and ${\rho _C} = t{r_{AB}}({\rho _{ABC}})$. Hence, the tripartite uncertainty can be expressed as:

20 $$\begin{aligned} \mathcal{U}= & {} S({\rho _{{\sigma _x}B}}) - S({\rho _B}) + S({\rho _{{\sigma _z}C}}) - S({\rho _C}) \nonumber \\= & {} {h_{bin}}\left( {{{1 - \sqrt{1 - 4\omega } } \over 2}} \right) \nonumber \\&- \sum \limits _{i = 6,7} {{h_{bin}}\left( {{{1 - 2{A_{ii}}} \over 2}} \right) }+ \sum \limits _{i = 4,6,7} {{A_{ii}}{{\log }_2}{A_{ii}} + } {3 \over 2}, \end{aligned}$$

where $\omega = {A_{11}}{A_{44}} + {A_{44}}{A_{66}} + {A_{11}}{A_{77}} + {A_{66}}{A_{77}} - ({A_{46}}A_{17}^*)({A_{17}}A_{46}^*)$, and ${h_{bin}}(\mu ) = - \mu {\log _2}\mu - (1 - \mu ){\log _2}(1 - \mu )$ is the binary entropy function.

With some simplifications and the fact that the complementarity of any pair of Pauli observables is ${c=\frac{1}{2}}$, Ming’s lower bound $U_M$ takes the following form:

21 $$\begin{aligned} {U_M}= & {} 1 + \max \{ 0,\Delta ^{(xz)} \},\nonumber \\ \Delta ^{(xz)}= & {} 1 + S({\rho _{AB}}) + S({\rho _{AC}}) - S({\rho _{{\sigma _x}C}}) - S({\rho _{{\sigma _z}B}}) \end{aligned}$$

The von Neumann entropies $S(\rho )$ could be calculated by using the eigenvalues (noted by $\lambda _i$) of $\rho$ as follows:

22 $$\begin{aligned} S({\rho _\alpha })= & {} \sum \limits _{i = 1}^4 {\lambda _i^\alpha {{\log }_2}\lambda _i^\alpha } ,\quad \quad (\alpha = AB,AC),\nonumber \\ S({\rho _{{\sigma _x}C}})= & {} {h_{bin}}\left( {{{1 - \sqrt{1 - 4\varpi } } \over 2}} \right) + 1,\nonumber \\ S({\rho _{{\sigma _z}B}})= & {} {1 \over 2} - \sum \limits _{i = 4,6,7} {{A_{ii}}{{\log }_2}{A_{ii}}}, \end{aligned}$$

where $$\lambda _{1,2}^{AB} = {1 \over 2}[{A_{11}} + {A_{77}} \pm \sqrt{{{({A_{11}} - {A_{77}})}^2} + 4{{\left| {{A_{17}}} \right| }^2}} ]$$, $$\lambda _{3,4}^{AB} = {1 \over 2}[{A_{44}} + {A_{66}} \pm \sqrt{{{({A_{44}} - {A_{66}})}^2} + 4{{\left| {{A_{46}}} \right| }^2}} ]$$, $$\lambda _{1,2}^{AC} = {1 \over 2}[{A_{11}} + {A_{66}} \pm \sqrt{{{({A_{11}} - {A_{66}})}^2} + 4{{\left| {{A_{16}}} \right| }^2}} ]$$, $$\lambda _{3,4}^{AC} = {1 \over 2}[{A_{44}} + {A_{77}} \pm \sqrt{{{({A_{44}} - {A_{77}})}^2} + 4{{\left| {{A_{47}}} \right| }^2}} ]$$, and $$\varpi = {A_{11}}{A_{44}} + {A_{11}}{A_{66}} + {A_{44}}{A_{77}} + {A_{66}}{A_{77}} - ({A_{47}}A_{16}^*)({A_{16}}A_{47}^*)$$.

For the same observables, $\sigma _x$ and $\sigma _z$, Dolatkhah’s lower bound $U_D$ can be written as:

23 $$\begin{aligned} {U_D}= & {} 1 + {{S({\rho _{AB}}) + S({\rho _{AC}}) - S({\rho _B}) - S({\rho _C})} \over 2} + \max \{ 0,\delta ^{(xz)} \},\nonumber \\ \delta ^{(xz)}= & {} {{2S({\rho _A}) + S({\rho _B}) + S({\rho _C}) - [S({\rho _{AB}}) + S({\rho _{AC}})]} \over 2} \nonumber \\&- [\mathrm{{I(X:B) + I(Z:C)}}], \end{aligned}$$

where

24 $$\begin{aligned} \mathrm{{I}}(\mathrm{{X}}:\mathrm{{B}})= & {} {h_{bin}}\left( {{{1 - 2{A_{66}}} \over 2}} \right) - {h_{bin}}\left( {{{1 - \sqrt{1 - 4\omega } } \over 2}} \right) ,\nonumber \\ \mathrm{{I}}(\mathrm{{Z}}:\mathrm{{C}})= & {} \sum \limits _{i = 4,7} {{h_{bin}}\left( {{{1 - 2{A_{ii}}} \over 2}} \right) } + \sum \limits _{i = 1,4,6,7} {{A_{ii}}} \log {A_{ii}}. \end{aligned}$$

Again, the von Neumann entropies of the reduced density matresices ${\rho _A}$, ${\rho _B}$, and ${\rho _C}$ are:

25 $$\begin{aligned} S({\rho _A})= & {} {h_{bin}}\left( {1/2 - {A_{44}}} \right) ,\quad S({\rho _B}) = {h_{bin}}\left( {1/2 - {A_{66}}} \right) ,\nonumber \\ S({\rho _C})= & {} {h_{bin}}\left( {1/2 - {A_{77}}} \right) . \end{aligned}$$

**Figure 1:**
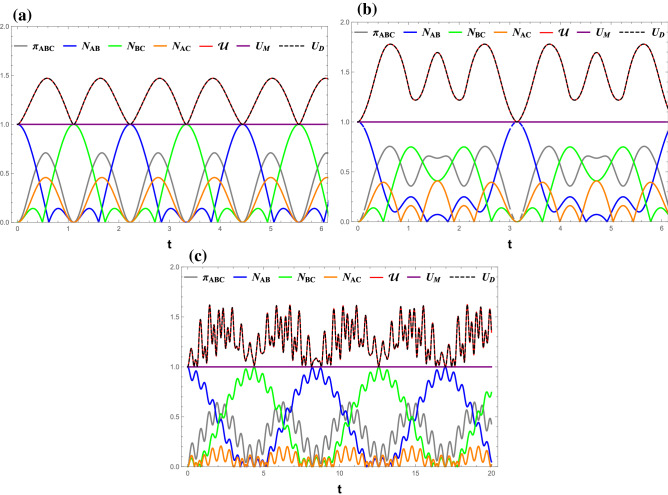
(Color online) Time evolutions of $\mathcal{U}$ (red line), $U_M$ (purple line), $U_D$ (black dashed), $\pi _{ABC}$ (gray line), $N_{AB}$ (blue line), $N_{BC}$ (green line), and $N_{AC}$ (orange line) under the measurement ($\sigma _x$, $\sigma _z$). (**a**) $J_z$ = 0, (**b**) $J_z$ = 1, and (**c**) $J_z$ = 5. All Figs. $\gamma$ = 0, *B* = 0.

**Figure 2:**
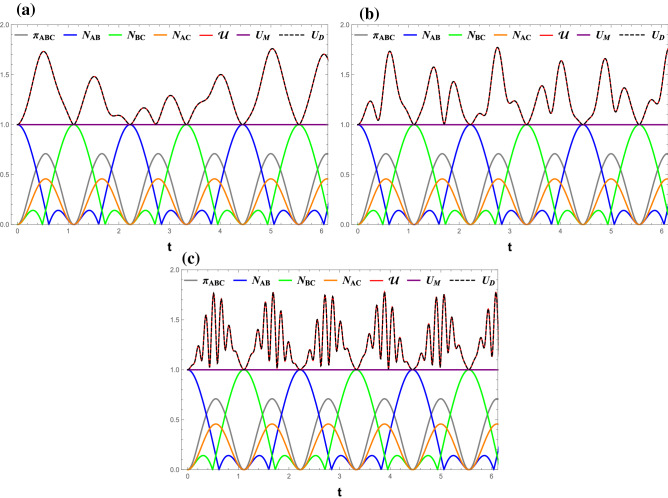
(Color online) Time evolutions of $$ \mathcal{U} $$ (red line), $$ U_M $$ (purple line), $$ U_D $$ (black dashed), $$ \pi _{ABC} $$ (gray line), $$ N_{AB} $$ (blue line), $$ N_{BC} $$ (green line), and $$ N_{AC} $$ (orange line) under the measurement ($$\sigma _x$$, $$\sigma _z$$). (**a**) *B* = 1, (**b**) *B* = 3, and (**c**) *B* = 10. All Figs. $$\gamma $$ = 0, $$J_z $$ = 0.

**Figure 3:**
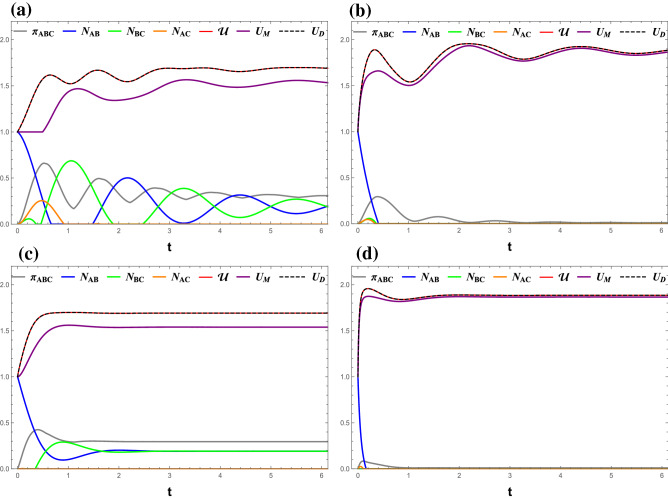
(Color online) Time evolutions of $$ \mathcal{U} $$ (red line), $$ U_M $$ (purple line), $$ U_D $$ (black dashed), $$ \pi _{ABC} $$ (gray line), $$ N_{AB} $$ (blue line), $$ N_{BC} $$ (green line), and $$ N_{AC} $$ (orange line) under the measurement ($$\sigma _x$$, $$\sigma _z$$). (**a**) $$\gamma $$ = 0.1, *B* = 0, (**b**) $$\gamma $$ = 0.1, *B* = 2, and (**c**) $$\gamma $$ = 0.5, *B* = 0, and (**d**) $$\gamma $$ = 0.5, *B* = 2. All Figs. $$J_z $$ = 0.

The great relevance between bipartite QMA-EUR and quantum correlation^19–28^ encouraged us to include entanglement in our study, by considering the residual entanglement to quantify simultaneous entanglement between all qubits in a multi-qubit system. For a three-qubit system, the residual entanglement can be expressed as:

26 $$\begin{aligned} {\pi _{ABC}}= & {} {1 \over 3}({\pi _A} + {\pi _B} + {\pi _C}),\nonumber \\ {\pi _A}= & {} N_{A(BC)}^2 - N_{AB}^2 - A_{AC}^2,\nonumber \\ {\pi _B}= & {} N_{B(AC)}^2 - N_{BA}^2 - A_{BC}^2,\nonumber \\ {\pi _C}= & {} N_{C(AB)}^2 - N_{CA}^2 - A_{CB}^2, \end{aligned}$$

where the negativity of tripartite and bipartite systems can be expressed, respectively, as ${N_{\alpha \beta }} = \left\| {\rho _{\alpha \beta }^{{T_\alpha }}} \right\| - 1$ and ${N_{\alpha (\beta \gamma )}} = \left\| {\rho _{\alpha \beta \gamma }^{{T_\alpha }}} \right\| - 1$, $\Vert \rho \Vert = tr[\sqrt{\rho {\rho ^\dag }} ]$ is the trace norm of $\rho$, ${\rho ^{{T_\alpha }}}$ represents the partial transpose of $\rho$ with respect to the qubit $\alpha$.

Figure 1 displays the dynamics of $\mathcal{U}$, $U_M$, and $U_D$ for the case without decoherence, $\gamma =0$. The residual entanglement, $\pi _{ABC}$, and bipartite entanglement, $N_{\alpha \beta }$, are also included.

One can observe that all quantities, except $U_M$, oscillate periodically with the growth of time *t*. The three bipartite negativities, $N_{AB}$, $N_{BC}$, and $N_{AC}$ exhibit different behaviors in such a way that nearest-neighbor entanglements, $N_{AB}$ and $N_{BC}$, evolve oppositely, namely if one is maximum, the other is zero. In contrast, the next-to-nearest neighbor entanglement, $N_{AC}$, is modulated the same as the residual entanglement ($\pi _{ABC}$) with a different amplitude. On the other hand, the time variations of $\mathcal{U}$, $U_M$, and $U_D$ can be summarized in the following points:

- Regardless of the $J_z$ value, the entropic uncertainty perfectly corresponds to the residual entanglement, indicating that the uncertainty in the measurement outcome increases with the overall entanglement of the system.
- Interestingly, it can be observed that the oscillation of $U_D$ is completely identical to that of $\mathcal{U}$, while $U_M$ freezes at a lower value 1. This indicates that Dolatkhah’s lower bound is tighter than Ming’s lower bound and can be used to express entropic uncertainty $\mathcal{U}$. This conclusion is confirmed for various $J_z$ values when ($\sigma _x$, $\sigma _z$) is the pair of the incompatible observables as shown in Figs. 2 and 3.
- $\mathcal{U}$, and therefore $U_D$, reaches its minima if either pair of nearest-neighbor qubits is maximally entangled. Their oscillation frequency increases with $J_z$.

The dynamics of all quantities in various magnetic fields is shown in Fig. 2. Despite the fact that the quantum entanglement does not change with *B*, the fluctuation of the entropic uncertainty $\mathcal{U}$ increases significantly with *B*. This indicates that the entropic uncertainty is not necessary to be synchronized with the entanglement in all circumstances. In addition, the freezing of $U _{M}=1$ was not violated by the presence of a magnetic field, similar to what has been found in Fig. 1. Also, the assertion that minimal $\mathcal{U} = U_D = 1$ is associated with the maximum entanglement between one of the nearest-neighbor qubit pair remains valid.

Our discussion will now focus on the decoherence effects on the tripartite entropic uncertainty and its lower bounds. As what can be easily seen in Fig.  3, the decoherence eliminates the regular oscillatory behaviors of $\mathcal{U}$, $U_D$, and all entanglement measures after some time related to $\gamma$. Also, the presence of the intrinsic decoherence breaks the frozen behavior of $U_M$, which shows a damped oscillation similar to that of $\mathcal{U}$ and $U_D$. There are some other findings similar to what aforementioned including: $\mathcal{U}$ and $U_D$ are identical, $\mathcal{U}$ and $\pi _{ABC}$ are synchronized, and the minimum of $\mathcal{U}$ corresponds to the maximum of $N _{AB}$ or $N _{BC}$ Finally, it can be concluded that $\mathcal{U}$, $U_M$ and $U_D$ evolve together into the same steady maxima as there is diminishing quantum entanglement between the qubits resulting from a greater *B* and $\gamma$ as shown in Fig. 3b,d.

The persistent overlapping between $\mathcal{U}$ and $U_D$ illustrated in the previous figures can be shown as follows: According to Eq. (10), Dolatkhah’s bound can be expressed as:

27 $$\begin{aligned} {U_D} = {q_{\mathrm{{MU}}}} + {{S(\left. A \right| B) + S(\left. A \right| C)} \over 2} + \delta , \end{aligned}$$

and can be further simplified as (Eq. 23):

28 $$\begin{aligned} {U_D} = 1 + S({\rho _A}) - [I(X:B) + I(Z:C)]. \end{aligned}$$

With the measurement pair ($\sigma _x$, $\sigma _z$), the information amount that Alice shares with Bob and Charlie, *I*(*X* : *B*) and *I*(*Z* : *B*) are explicitly given by Eq. (24). Inserting these values and ${A_{11}} = {1 \over 2}$ into Eq. (28), one can finally get:

29 $$\begin{aligned} {U_D}= & {} {h_{bin}}\left( {{{1 - \sqrt{1 - 4\omega } } \over 2}} \right) - \sum \limits _{i = 6,7} {{h_{bin}}\left( {{{1 - 2{A_{ii}}} \over 2}} \right) }\nonumber \\&+ \sum \limits _{i = 4,6,7} {{A_{ii}}{{\log }_2}{A_{ii}} + } {3 \over 2}\nonumber \\= & {} \mathcal{U}. \end{aligned}$$

### QMA-EUR for ($\sigma _x$, $\sigma _y$)-measurement pair:

In this section, we consider that Alice chooses the observables pair $(\sigma _x$ , $\sigma _y$). The tripartite uncertainty in this scenario can be expressed as:

30 $$\begin{aligned} \mathcal{U}= & {} S({\rho _{{\sigma _x}B}}) - S({\rho _B}) + S({\rho _{{\sigma _y}C}}) - S({\rho _C})\nonumber \\= & {} \sum \limits _{\alpha = \omega ,\varpi } {{h_{bin}}\left( {{{1 - \sqrt{1 - 4\alpha } } \over 2}} \right) } - \sum \limits _{i = 6,7} {{h_{bin}}\left( {{{1 - 2{A_{ii}}} \over 2}} \right) } + 2, \end{aligned}$$

where $\omega$ and $\varpi$ are the same as those in Eqs. (20) and (21), respectively.

**Figure 4:**
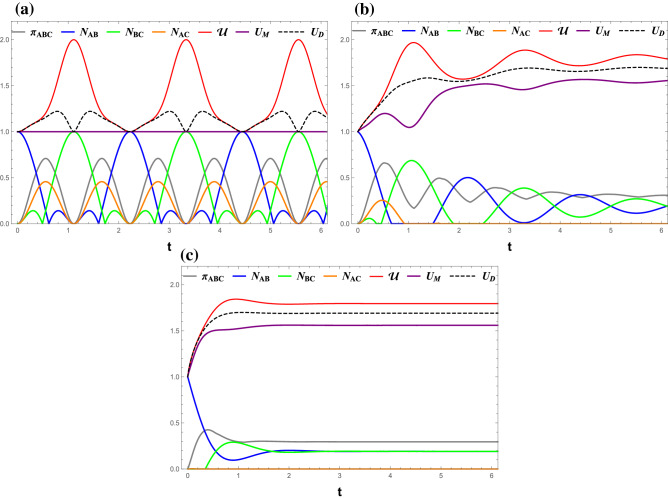
(Color online) Time evolutions of $$ \mathcal{U} $$ (red line), $$ U_M $$ (purple line), $$ U_D $$ (black dashed), $$ \pi _{ABC} $$ (gray line), $$ N_{AB} $$ (blue line), $$ N_{BC} $$ (green line), and $$ N_{AC} $$ (orange line) under the measurement ($$\sigma _x$$, $$\sigma _y$$). (**a**) $$ \gamma $$ = 0, (**b**) $$ \gamma $$ = 0.1, and (**c**) $$ \gamma $$ = 0.5 All Figs. $$J_z $$ = 0, *B* = 0.

**Figure 5:**
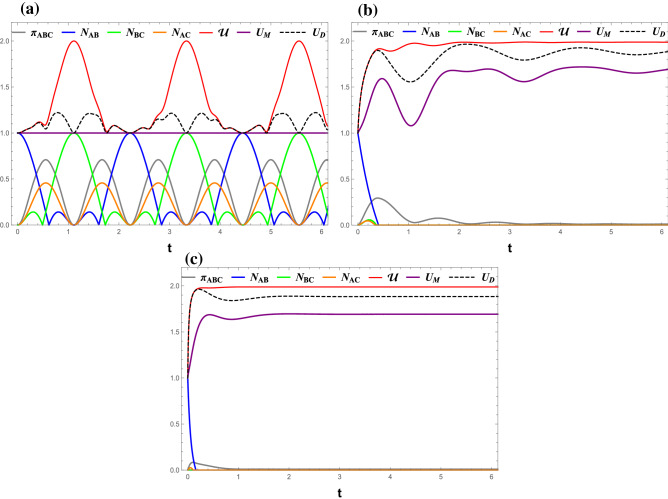
(Color online) Time evolutions of $$ \mathcal{U} $$ (red line), $$ U_M $$ (purple line), $$ U_D $$ (black dashed), $$ \pi _{ABC} $$ (gray line), $$ N_{AB} $$ (blue line), $$ N_{BC} $$ (green line), and $$ N_{AC} $$ (orange line) under the measurement ($$\sigma _x$$, $$\sigma _y$$). (**a**) $$ \gamma $$ = 0, (**b**) $$ \gamma $$ = 0.1, and (**c**) $$ \gamma $$ = 0.5. All Figs. $$J_z $$ = 0, *B* = 2.

On the other hand, Ming’s and Dolatkhah’s lower bounds remain the same forms as those for ($\sigma _x$, $\sigma _z$) measurement pair by simply replacing ${\Delta ^{(xz)}}$ and ${\delta ^{(xz)}}$ by ${\Delta ^{(xy)}}$ and ${\delta ^{(xy)}}$, respectively:

31 $$\begin{aligned} {\Delta ^{(xy)}}= & {} 1 + S({\rho _{AB}}) + S({\rho _{AC}}) - S({\rho _{{\sigma _x}C}}) - S({\rho _{{\sigma _y}B}}),\nonumber \\ {\delta ^{(xy)}}= & {} {{2S({\rho _A}) + S({\rho _B}) + S({\rho _C}) - [S({\rho _{AB}}) + S({\rho _{AC}})]} \over 2}\nonumber \\&- [\mathrm{{I}}(\mathrm{{X}}:\mathrm{{B}})\mathrm{{ + I}}(\mathrm{{Y}}:\mathrm{{C}})], \end{aligned}$$

in which

32 $$\begin{aligned} S({\rho _{{\sigma _y}B}})= & {} {h_{bin}}\left( {{{1 - \sqrt{1 - 4\omega } } \over 2}} \right) + 1,\nonumber \\ \mathrm{{I}}(\mathrm{{Y}}:\mathrm{{C}})= & {} S({\rho _C}) - {h_{bin}}\left( {{{1 - \sqrt{1 - 4\varpi } } \over 2}} \right) , \end{aligned}$$

Other von Neumann entropies and the Holevo quantity $\mathrm{{I}}(\mathrm{{X}}:\mathrm{{B}})$ are the same as those in the case of ($\sigma _x$, $\sigma _z$) pair.

The results for $$B=0$$ and $$B=2$$ are respectively shown in Figs. 4, 5. Comparing with the results for ($$\sigma _x$$, $$\sigma _z$$) case shown in Figs. 1, 2 and 3 one can easily conclude that the tripartite QMA-EUR and the two bounds do depend on the choice of measurement pair. The only exception $$ U_M=1 $$ occurs in the special case $$ \gamma =0 $$ regardless of which pair is chosen. In addition, unlike the results for ($$\sigma _x$$, $$\sigma _z$$), $$ \mathcal{U}$$ and $$U_D $$ are no longer the same but $$ \mathcal{U} \ge U_D $$ still holds for ($$\sigma _x$$, $$\sigma _y$$).

Figure 4 shows the dynamics of all quantities for various decoherence rates $$ \gamma $$ at $$ B=J_z=0 $$. It can be seen that the entropic uncertainty $$ \mathcal{U} $$ is related to $$ N _{AB} $$ and $$ N _{BC} $$ rather than $$ N _{AC} $$ and $$ \pi _{ABC}$$. The minimum of $$ \mathcal{U} $$ is associated with the maximum of $$ N _{AB} $$ and the minimum of $$ N _{BC} .$$ A closer inspection reveals that there is a tiny anomaly in the $$ \gamma =0 $$ case (Fig. 4a), in which $$ N _{AB}$$ exhibits one more peaks per period than $$\mathcal{U}$$.

The importance of nearest-neighbor entanglement asserts that the best guess on the measurement outcomes can be obtained when Alice is maximally entangled with Bob but not with Charlie. We can also state that the measurement accuracy is affected by the choice of measured observables, since the best guess occurs when either nearest neighbors are maximally entangled for the ($\sigma _x$, $\sigma _z$) choice, but not for the other.

The presence of the magnetic field *B* changes the way the $\mathcal{U}$, $U_M$, and $U_M$ oscillate, as shown in Fig. 5. It also assists the decay of entanglement between system components with a finite $\gamma$, thus shortens the time $\mathcal{U}$, $U_M$, and $U_M$ saturate to a fixed maximum compared to that for $B=0$.

Regardless of the various cases in our study, Dolatkhah’s bound is tighter than Ming’s and the inequality $2 \ge \mathcal{U} \ge U_D \ge U_M \ge 1$ always holds.

## Conclusions

We have investigated the dynamical characteristic of the tripartite QMA-EUR and its lower bounds in a three-qubit Heisenberg XXZ spin chain under intrinsic decoherence. The relationship between tripartite uncertainty and quantum entanglement between system components has been also investigated using tripartite and bipartite negativities. The results clearly confirm the dependence of the tripartite uncertainty on the choice of observable pair that Alice would measure. We showed that an inequality for the tripartite uncertainty, $2 \ge \mathcal{U} \ge U_D \ge U_M \ge 1$ always holds and Dolatkhah’s lower bound $U_D$ is identical to the tripartite uncertainty, $\mathcal{U} = U_D$, with the complementary measurements pair ($\sigma _x$, $\sigma _z$). When there is no intrinsic decoherence, Ming’s lower bound $U_M=1$ is always fixed as the time evolves with any choice of Pauli measurement pair. We have also verified that the connection between uncertainty and the quantum entanglement between the system components would change with the choice of incompatible observables: for the pair ($\sigma _x$, $\sigma _z$), the best measurement accuracy occurs when the nearest neighbors are maximally entangled, while for ($\sigma _x$, $\sigma _y$) the best measurement accuracy can be obtained when Alice is maximally entangled with Bob. Our results provide a better understanding of the lower bounds of the tripartite QMA-EUR which are crucial in improving quantum measurements in quantum information processing.

## References

[1] Heisenberg W (1985). Über den anschaulichen inhalt der quantentheoretischen kinematik und mechanik. Original Scientific Papers Wissenschaftliche Originalarbeiten, pp 478–504.

[2] Kennard EH (1927). Zur quantenmechanik einfacher bewegungstypen. Z. Phys..

[3] Robertson HP (1929). The uncertainty principle. Phys. Rev..

[4] Kraus K (1987). Complementary observables and uncertainty relations. Phys. Rev. D.

[5] Maassen H, Uffink JBM (1988). Generalized entropic uncertainty relations. Phys. Rev. Lett..

[6] Berta M, Christandl M, Colbeck R, Renes JM, Renner R (2010). The uncertainty principle in the presence of quantum memory. Nat. Phys..

[7] Bai X-M, Xue N-T, Liu N, Li J-Q, Liang J-Q (2019). The entropic uncertainty relation for two qubits in the cavity-based architecture. Ann. Phys..

[8] Adabi F, Salimi S, Haseli S (2016). Tightening the entropic uncertainty bound in the presence of quantum memory. Phys. Rev. A.

[9] Dupuis F, Fawzi O, Wehner S (2014). Entanglement sampling and applications. IEEE Trans. Inf. Theory.

[10] Konig R, Wehner S, Wullschleger J (2012). Unconditional security from noisy quantum storage. IEEE Trans. Inf. Theory.

[11] Vittorio G, Seth L, Lorenzo M (2011). Advances in quantum metrology. Nat. Photon..

[12] Chang-shui Y (2017). Quantum coherence via skew information and its polygamy. Phys. Rev. A.

[13] Vallone G, Marangon DG, Tomasin M, Villoresi P (2014). Quantum randomness certified by the uncertainty principle. Phys. Rev. A.

[14] Cao Z, Zhou H, Yuan X, Ma X (2016). Source-independent quantum random number generation. Phys. Rev. X.

[15] Coles PJ, Piani M (2014). Complementary sequential measurements generate entanglement. Phys. Rev. A.

[16] Ming-Liang H, Fan H (2013). Upper bound and shareability of quantum discord based on entropic uncertainty relations. Phys. Rev. A.

[17] Mohamed A-BA, Eleuch H, Ooi CHR (2019). Non-locality correlation in two driven qubits inside an open coherent cavity: Trace norm distance and maximum bell function. Sci. Rep..

[18] Chen X-Y, Jiang L-Z, Zhu-An X (2018). Precise detection of multipartite entanglement in four-qubit Greenberger–Horne–Zeilinger diagonal states. Front. Phys..

[19] Yao Y-B, Wang D, Ming F, Ye L (2020). Dynamics of the measurement uncertainty in an open system and its controlling. J. Phys. B Atom. Mol. Opt. Phys..

[20] Wang D, Ming F, Huang A-J, Sun W-Y, Ye L (2017). Entropic uncertainty for spin-1/2 xxx chains in the presence of inhomogeneous magnetic fields and its steering via weak measurement reversals. Laser Phys. Lett..

[21] Wang D, Ming F, Ming-Liang H, Ye L (2019). Quantum-memory-assisted entropic uncertainty relations. Ann. Phys..

[22] Wang D, Shi W-N, Hoehn RD, Ming F, Sun W-Y, Ye L, Kais S (2018). Probing entropic uncertainty relations under a two-atom system coupled with structured bosonic reservoirs. Quantum Inf. Process..

[23] Ming F, Wang D, Shi W-N, Huang A-J, Ming-Ming D, Sun W-Y, Ye L (2018). Exploring uncertainty relation and its connection with coherence under the Heisenberg spin model with the dzyaloshinskii-moriya interaction. Quantum Inf. Process..

[24] Zhang Z-Y, Wei DX, Liu J-M (2018). Entropic uncertainty relation of a two-qutrit Heisenberg spin model in nonuniform magnetic fields and its dynamics under intrinsic decoherence. Laser Phys. Lett..

[25] Huang Z (2018). Quantum-memory-assisted entropic uncertainty in spin models with Dzyaloshinskii–Moriya interaction. Laser Phys. Lett..

[26] Abdelghany RA, Mohamed A-BA, Tammam M, Obada A-SF (2020). Dynamical characteristic of entropic uncertainty relation in the long-range ising model with an arbitrary magnetic field. Quantum Inf. Process..

[27] Zidan N (2020). Entropic uncertainty in spin xy model with long-range interactions. Entropy.

[28] Yang Y-Y, Sun W-Y, Shi W-N, Ming F, Wang D, Ye L (2019). Dynamical characteristic of measurement uncertainty under Heisenberg spin models with dzyaloshinskii-moriya interactions. Front. Phys..

[29] He J, Ding ZY, Shi JD, Liu CC, Wu T (2020). Tighter bound of entropic uncertainty under the Unruh effect. Ann. Phys..

[30] Yang Y-Y, Ye L, Wang D (2020). Measurement uncertainty and its connection to quantum coherence in an inertial Unruh–Dewitt detector. Ann. Phys..

[31] Wang D, Ming F, Song X-K, Ye L, Chen J-L (2020). Entropic uncertainty relation in neutrino oscillations. Eur. Phys. J. C.

[32] Chen M-N, Wang D, Ye L (2019). Characterization of dynamical measurement’s uncertainty in a two-qubit system coupled with bosonic reservoirs. Phys. Lett. A.

[33] Ming F, Song X-K, Ling J, Ye L, Wang D (2020). Quantification of quantumness in neutrino oscillations. Eur. Phys. J. C.

[34] Juju H, Ji Y (2020). Manipulating of the entropic uncertainty in open quantum system: Via quantum-jump-based feedback control. Int. J. Theor. Phys..

[35] Haseli S (2020). Quantum-memory-assisted entropic uncertainty relation with moving quantum memory inside a leaky cavity. Eur. Phys. J. Plus.

[36] Renes JM, Boileau J-C (2009). Conjectured strong complementary information tradeoff. Phys. Rev. Lett..

[37] Ming F, Wang D, Fan X-G, Shi W-N, Ye L, Chen J-L (2020). Improved tripartite uncertainty relation with quantum memory. Phys. Rev. A.

[38] Dolatkhah H, Haseli S, Salimi S, Khorashad AS (2020). Tightening the tripartite quantum-memory-assisted entropic uncertainty relation. Phys. Rev. A.

[39] Glaser U, Büttner H, Fehske H (2003). Entanglement and correlation in anisotropic quantum spin systems. Phys. Rev. A.

[40] Loss D, DiVincenzo DP (1998). Quantum computation with quantum dots. Phys. Rev. A.

[41] Burkard G, Loss D, DiVincenzo DP (1999). Quantum computation with quantum dots. Phys. Rev. B.

[42] Toskovic R, van den Berg R, Spinelli A, Eliens IS, van den Toorn B, Bryant B, Caux J-S, Otte AF (2016). Atomic spin-chain realization of a model for quantum criticality. Nat. Phys..

